# Patient Age Is Significantly Related to Distant Metastasis of Papillary Thyroid Microcarcinoma

**DOI:** 10.3389/fendo.2021.748238

**Published:** 2021-12-23

**Authors:** Hui Huang, Siyuan Xu, Xiaolei Wang, Shaoyan Liu, Jie Liu

**Affiliations:** Department of Head and Neck Surgical Oncology, National Cancer Center/National Clinical Research Center for Cancer/Cancer Hospital, Chinese Academy of Medical Sciences and Peking Union Medical College, Beijing, China

**Keywords:** papillary microcarcinoma, thyroid, distant metastasis, age, survive

## Abstract

**Objective:**

Distant metastasis in papillary thyroid microcarcinoma (PTMC) is rare but fatal, and its relationship with patient age remains unclear. The objective of this study was to examine the association between age at diagnosis and metachronous distant metastasis in PTMC.

**Methods:**

Consecutive patients who underwent thyroidectomy for PTC measuring 10 mm or less at a tertiary hospital from January 2000 to December 2016 were enrolled. Patients who had evidence of distant metastasis at diagnosis or underwent postoperative radioiodine (RAI) ablation were excluded. A Cox proportional hazards model with restricted cubic splines (RCS) was applied to examine the association between age at diagnosis and distant metastasis.

**Results:**

A total of 4,749 patients were evaluated. The median age was 44 years (range, 8–78 years), and 3,700 (78%) were female. After a median follow-up of 65 months, 21 distant metastases (20 lung, 1 liver) were recognized. A univariate Cox proportional model using a 5-knot RCS revealed a significant overall (*p* = 0.01) and a potential nonlinear association (*p* = 0.08) between distant metastasis and age at diagnosis. In multivariate analysis, age at diagnosis, extrathyroidal extension (ETE), and lymph node metastasis (pN+) were independent risk factors for distant metastasis. Compared with the middle-aged group (30–45 years old), younger and older patients had a higher risk of distant metastasis [HR, 95% CI, *p*-value, age ≤ 30, 4.54 (0.91–22.60), 0.06, age > 45, 6.36 (1.83–22.13), <0.01].

**Conclusion:**

Age at diagnosis is associated with metachronous distant metastasis of PTMC, and patients with younger or older age have a higher risk of distant metastasis than middle-aged patients.

## Introduction

Papillary thyroid microcarcinoma (PTMC) is defined as a small papillary thyroid cancer (PTC) measuring 10 mm or less (1). PTMC has an indolent nature and very little risk of disease-related mortality; thus, it is controversial whether the tumor should be immediately managed after diagnosis. In recent years, PTMC has been considered to warrant a more conservative strategy for both diagnosis and treatment. For instance, active surveillance (AS), rather than immediate intervention, is becoming a popular and well-accepted approach (2–5). The decision-making regarding AS or immediate surgery generally depends on the tumor location, extrathyroidal extension (ETE), and presence of lymph node metastasis (LNM). According to several current guidelines of AS for PTMC, indications for immediate surgery include the presence of lymph node or distant metastasis, aggressive subtypes, suspicious invasion of important structures (recurrent laryngeal nerve or trachea) of the neck or a tumor located near these structures (6, 7). Although age < 20 years also has recommendation for surgery by Consensus Statements from the Japan Association of Endocrine Surgery Task F,orce (7), patient age is not considered a strong factor when considering AS. Nonetheless, how to differentiate the small proportion of high-risk patients according to the staging system of the whole PTC is still concerning, and patient age and distant metastasis are the two most important prognostic factors related to disease-specific mortality. Although rare, distant metastasis is also a possible fate of PTMC, and the association of distant metastasis with age remains unclear. The objective of the present study was to examine the association between age and metachronous distant metastasis in patients with PTMC, which may provide additional information for considering treatment strategy.

## Materials and Methods

This study was approved by the Ethics Committee of the Cancer Hospital, Chinese Academy of Medical Sciences. Informed consent was obtained at the time of surgery with surgical consent for the general use of clinical information for future studies. This study followed the Strengthening the Reporting of Observational Studies in Epidemiology (STROBE) reporting guidelines for observational studies. Consecutive patients who underwent thyroidectomy for PTC measuring 10 mm or less at a tertiary hospital from January 2000 to December 2016 were retrospectively analyzed. Data analysis was performed from May to June 2021. Variables such as age at diagnosis, sex, tumor characteristics, and treatment modalities were obtained from the medical records. Patients who had evidence of distant metastasis at diagnosis, underwent postoperative radioiodine ablation, or followed up less than 12 months were excluded.

Primary size, ETE, and LNM were defined by postoperative pathology. LNM was considered negative if no lymph nodes were examined. Clinical positive node was judged mainly with ultrasound; specific features such as short axis >5 mm, presence of cystic areas, and presence of hyperechogenic punctuations representing either colloid or microcalcifications are considered as clinical positive lymph node as well as positive FNA or Tg washout. Surgeries for primary tumors included lobectomy and total thyroidectomy; central compartment dissection was considered for clinical positive neck or patients with risk factors (ETE, larger primary) and therapeutic lateral neck dissection was performed in cases with standard indications. Survival outcomes were determined by medical records in combination with telephone follow-up. Local and regional recurrences were defined as structural disease as judged by either cytology or pathology. Distant metastasis was defined by chest computed tomography (CT) or in combination with a whole-body scan using I^131^ after total thyroidectomy was performed.

The association of age at diagnosis and distant metastasis was assessed using a Cox proportional hazards model with restricted cubic spline (RCS), providing a flexible model to analyze nonlinear association between continuous (age) and categorical (distant metastasis) variables. We then estimated age corresponding to change points in the relative risk of distant metastasis. We applied a simulation approach to assess the change point based on regression splines and a segmented regression model. Stratification was performed according to the age change points. Kaplan–Meier curves and log rank tests were generated to compare the risks of distant metastasis between different age subgroups. All statistical analyses were conducted with the R package, version 3.6.2 (R Foundation for Statistical Computing, Vienna, Austria). *p* < 0.05 was considered statistically significant.

## Results

### Baseline Characteristics of the Patients

A total of 4,749 patients were enrolled between 2000 and 2016. The median age was 44 years (range, 8–78 years), and 3,700 (78%) were female. The mean primary tumor size was 0.64 cm, and ETE and pathological LNM were identified in 1,836 (39%) and 1,772 (37%) patients, respectively (**Table 1**). After a median follow-up of 65 months, 21 distant metastases (20 lung metastases, 1 liver metastasis) were recognized. The median age of 21 patients who underwent distant metastasis was 53 years (range, 24–73 years), the female/male ratio was 2, the mean primary tumor size was 0.82 (0.3–1.0) cm, and ETE and pathological LNM were identified in 12 (57.1%) and 15 (71.4%) patients, respectively (**Table 1**) The time of distant metastasis diagnosis was 32–110 (median 58) months after surgery. A total of 132 recurrences and 4 PTC-related deaths were identified during follow-up.

**Table 1:** Baseline characteristics of the study cohort.

Characteristic	Whole cohort *N* (%)	Patients with distant metastasis *N* (%)
**Total**	4,749	21
**Age (median ± SD)**	44 ± 14	53 ± 13
**Sex**		
Male	1,049 (22)	7 (33)
Female	3,700 (78)	14 (67)
**Mean primary size** cm	0.6	0.8
**Multifocality**	1,690 (36)	6 (29)
**Extrathyroidal extension**	1,836 (39)	12 (57)
**Gross extrathyroidal extension**	110 (2)	4 (19)
**Hashimoto thyroiditis**	1,051 (22)	1 (5)
**Clinical lymph node metastasis**	406 (9)	7 (33)
**Pathological lymph node metastasis**	1,772 (37)	15 (71)
pN1a	1,376 (29)	8 (38)
pN1b	396 (8)	7 (33)
**Extent of surgery**		
Lobectomy	3,153 (66)	15 (71)
Total thyroidectomy	1,596 (34)	6 (29)

### Age and Distant Metastasis

**Figure 1:**
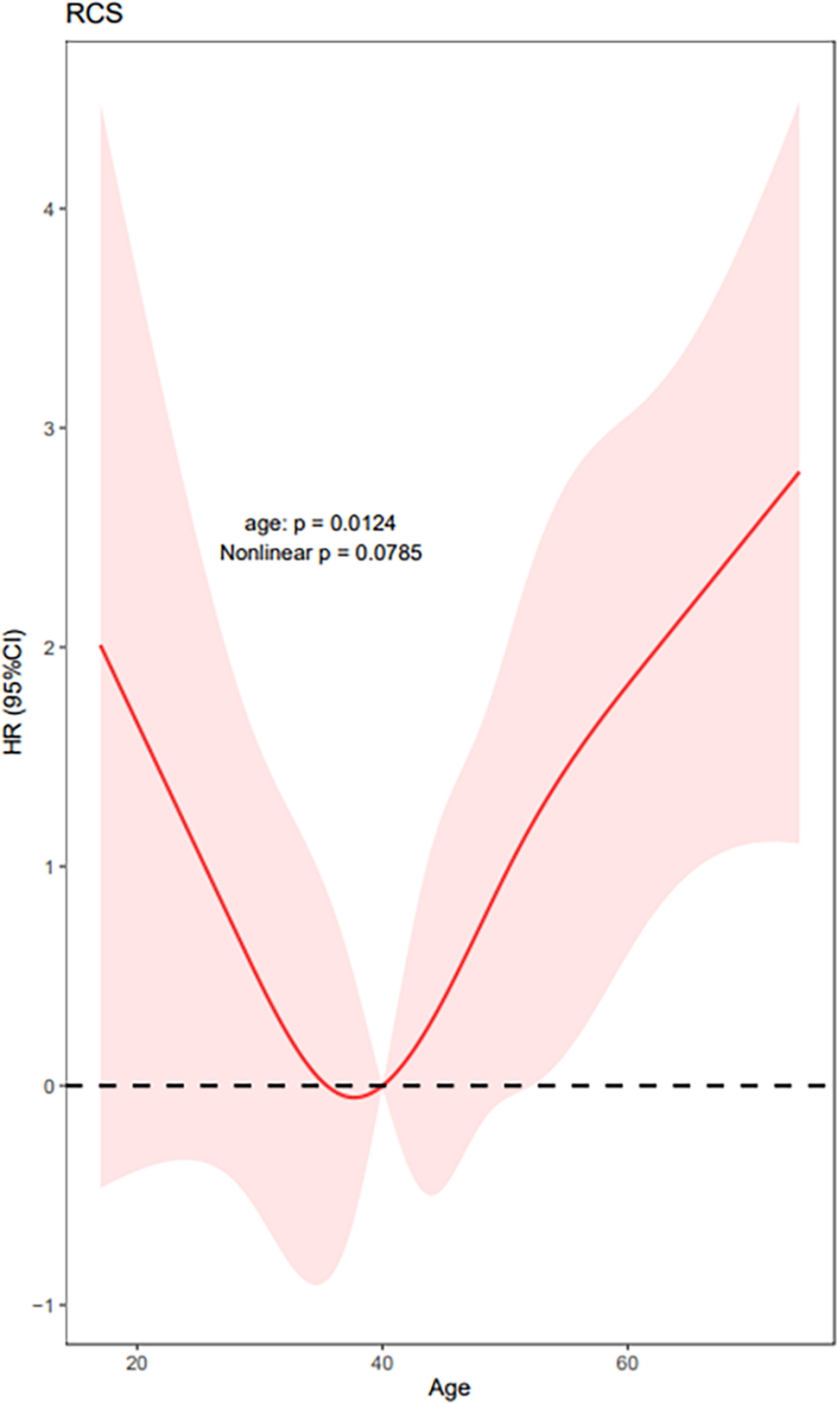
Univariate Cox proportional models by RCS show HRs of distant metastasis with increasing patient age at diagnosis. (Five knots were placed for RCS, age = 40 was selected as a reference point, and the lowest point estimated with simulation approach was 37.5 years; *p* for the overall model 0.01, *p* for nonlinear 0.08.).

**Figure 2:**
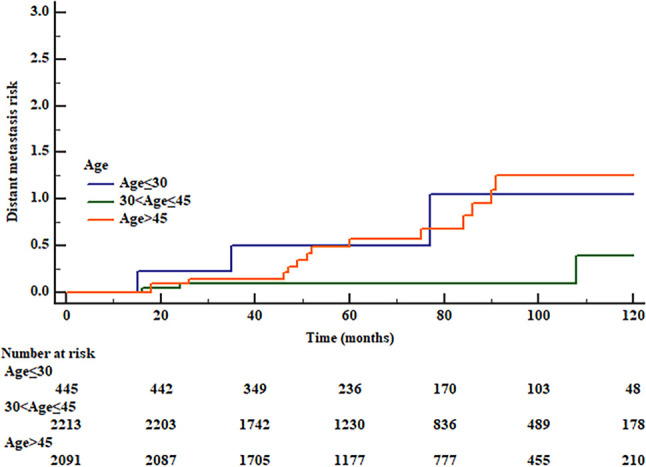
Risk of distant metastasis of different age groups by Kaplan–Meier curve. (0 reference group, age 30 < age ≤ 45; 1, age ≤ 30; 2, age > 45, log rank *p* = 0.02, 1 vs. 0 *p* = 0.25, 2 vs. 0 *p* = 0.001.).

We used RCS to create a flexible model and visualized the relationship of age and distant metastasis based on univariate Cox proportional models. Five knots were placed for RCS, age = 40 was selected as a reference point; the unadjusted HR with increasing age is shown in **Figure 1**. There was a significant overall association between the risk of distant metastasis and age at diagnosis (*p* = 0.01) in the univariable Cox proportional model by RCS, and a potential nonlinear association (*p* = 0.08) was also observed. The lowest point of the RCS curve was age = 37.5 years, and we then selected an interval of 30–45 (37.5 **±** 7.5) years old as the reference age group. HRs were estimated for age at diagnosis by using linear splines and different age intervals (30 < age ≤ 45, age ≤ 30, and age > 45, *n* = 2,213, 445, and 2,091, respectively) with and without adjustment for covariates. Univariate analysis indicated a significantly different risk of distant metastasis between the age subgroups (*p* = 0.02 age ≤ 30 vs. 30 < age ≤ 45 *p* = 0.25, age > 45 vs. 30 < age ≤ 45 *p* = 0.001) (**Figure 2**). According to multivariate analysis, age at diagnosis, ETE, and LNM (pN+) were independent risk factors for distant metastasis. Older age (age > 45) was significantly associated with an elevated risk of distant metastasis than aged 30–45 years [HR, 95% CI, 6.36 (1.83–22.13), *p* < 0.01], and risks of younger patients (age ≤ 30) and the reference group (30 < age ≤ 45) were close to the significant difference [HR, 95% CI, 4.54 (0.91–22.60), *p* = 0.06] (**Table 2**).

**Table 2:** Univariate and multivariate analyses of characteristics and distant metastasis risk by Cox regression.

Characteristics	Univariate analysis		Multivariate analysis	
	HR (95% CI)	*p*-value	HR (95% CI)	*p*-value
**Age**				
30 < age ≤ 45 (reference)	1		1	
≤30	4.88 (0.98–24.19)	0.05	4.54 (0.91–22.60)	0.06
>45	**5.08 (1.47–17.58)**	**0.01**	**6.36 (1.83–22.13)**	**0.003**
**Female**	0.84 (0.33–2.14)	0.72		
**Coexistent Hashimoto thyroiditis**	0.23 (0.03–1.74)	0.16	0.20 (0.03–1.56)	0.12
**Extra-thyroidal extension (ETE)**	**2.97 (1.18–7.45)**	**0.02**	**2.64 (1.06–6.59)**	**0.03**
**Clinical node metastasis (cN+)**	2.05 (0.72–5.85)	0.18		
**Multifocality**	0.51 (0.19–1.34)	0.17		
**Lymph node metastasis (pN+)**	**3.96 (1.32–11.88)**	**0.01**	**4.96 (1.85–13.28)**	**0.002**
**Total thyroidectomy**	**0.92 (0.36–2.40)**	0.87		

Bold values: factors significantly related to distant metastasis (p < 0.05).

## Discussion

Age is a significant prognostic factor in patients with PTC, and older age is associated with a significantly elevated risk of PTC-related death (8). However, as mortality caused by PTMC is especially rare, age is not considered as important as in those with larger PTCs. For instance, Ito et al. (9) insisted that in AS, younger age is related to greater progression risk than older age, and they concluded that AS was much safer for older patients. This is different from the situation in patients with large PTC, in whom older patients indicate a poor prognosis. In the present study, we detected a “U”-shaped association between age at diagnosis and distant metastasis in patients with PTMC. Compared with those in middle age, younger or older patients had an elevated risk of distant metastasis, especially when older than 45 years. The results indicate that age at diagnosis is also an important factor and should be included in the selection of treatment strategy. When aggressive clinicopathological factors are detected, young or old patients may need more systemic treatment (e.g., total thyroidectomy and RAI).

Most patients with PTMC have a favorable prognosis. Although rare, distant metastasis should be given more attention, as it may be fatal, and several previous studies have indicated some risk factors for distant metastasis. Giuseppe Mercante (10) analyzed 445 patients with PTMC and 4 distant metastases during follow-up. According to multivariate analysis, capsular invasion, ETE, and lymph node metastasis were independent risk factors for distant metastasis. Jeon et al. (11) studied 12 PTMCs with distant metastases (9 synchronous and 3 metachronous metastases) from 8,808 patients; they reported that the presence of extranodal extension and change to an aggressive pathologic subtype of metastatic lymph nodes were significantly associated with distant metastasis, which indicted an important association between the characteristics of regional lymph nodes and distant disease. Our analysis identified similar risk factors; moreover, our age stratification showed a significant association of age in multivariate analysis, constituting a novel risk factor for predicting distant metastasis risk in PTMC.

Treatment strategies may influence the presence of distant metastasis, especially when RAI is performed. In the present study, we selected patients who did not undergo RAI to avoid the influence of its prophylactic effect, and the cases in which distant metastasis developed may be under a natural history and be much more representative. Although patients who underwent AS were not included in this study, this had little influence on the results. As adverse factors were observed at diagnosis in all patients with distant metastasis, AS was not applicable. In addition, distant metastases in this analysis were identified at a relatively long time after surgery (30–110 months, median 58 months), and a longer follow-up time is needed to determine whether distant metastasis would be identified in patients who undergo nonsurgical treatment [AS- or ultrasound-guided radiofrequency ablation (12)]. On the other hand, surgical interventions should balance survival benefit and complication risk, and proper surgical approach should also be further clarified. For example, in some patients with PTC and clinical negative neck, prophylactic central neck dissection may result in elevated complication risk but little benefit (13). In regard to reducing distant metastasis risk, total thyroidectomy and subsequent RAI administration is required, but the benefit is unclear and needs further exploration.

There are a few limitations of the present study. First, the diagnosis of distant metastasis depended on chest CT in some patients with a residual thyroid lobe, and the diagnosis of early lung metastasis may be delayed. However, most distant metastases were confirmed with a subsequent whole-body I-131 scan after total thyroidectomy, and suspicious small lung nodules were followed to exclude metastasis. Second, as restricted by the target population of the study institute, adolescent patients were rare in the cohort (21 patients younger than 18 years), which may influence the statistical significance of the stratified analysis.

In conclusion, age at diagnosis is associated with distant metastasis in patients with PTMC, and young and old age are associated with an elevated risk compared with middle age.

## Data Availability Statement

The original contributions presented in the study are included in the article/supplementary material. Further inquiries can be directed to the corresponding authors.

## Ethics Statement

The studies involving human participants were reviewed and approved by the Ethic Committee of the Cancer Hospital, Chinese Academy of Medical Sciences. The patients/participants provided their written informed consent to participate in this study.

## Author Contributions

HH and SX participated in the design of the study, data collection, and paper writing. XW participated in the design of study and manuscript editing. SL participated in the data analysis and interpretation and manuscript review. JL participated in the design of the study and helped to revise the manuscript. All authors contributed to the article and approved the submitted version.

## Funding

The study was funded by Beijing Hope Run Special Fund of Cancer Foundation of China (Grant no.LC2018A26).

## Conflict of Interest

The authors declare that the research was conducted in the absence of any commercial or financial relationships that could be construed as a potential conflict of interest.

## Publisher’s Note

All claims expressed in this article are solely those of the authors and do not necessarily represent those of their affiliated organizations, or those of the publisher, the editors and the reviewers. Any product that may be evaluated in this article, or claim that may be made by its manufacturer, is not guaranteed or endorsed by the publisher.
